# Commentary: On the Emerging Role of Innate Lymphoid Cells in Bladder Cancer

**DOI:** 10.33696/cancerimmunol.6.093

**Published:** 2024

**Authors:** Zaineb Hassouneh, Gang Huang, Nu Zhang, Manjeet Rao, Neelam Mukherjee

**Affiliations:** 1 Department of Urology, University of Texas Health San Antonio (UTHSA), USA; 2 Department of Microbiology, Immunology & Molecular Genetics, UTHSA, USA; 3 Department of Cell Systems and Anatomy, UTHSA, USA; 4 Greehey Children’s Cancer Research Institute, UTHSA, USA

## Introduction

In the evolving landscape of bladder cancer (BCa) immunotherapy, this commentary offers a critical and timely reflection on a recent review that we published previously on targeting innate lymphoid cells (ILCs) in BCa. Even though BCa immunotherapy has traditionally focused on enhancing T-cell responses, the review explores the promising but underappreciated role of ILCs in BCa. From natural killer cells to the diverse ILC subtypes, these cells offer a dualistic impact on tumor progression and immune surveillance. By dissecting the review’s findings and integrating recent discoveries, this commentary emphasizes the potential of targeting ILCs to boost therapeutic efficacy and provide new avenues for BCa treatment. This analysis not only reaffirms the importance of integrating ILCs into immunotherapy strategies but also advocates for the application of these insights into novel immunotherapeutic strategies.

### The Twists and Turns of ILC Ontogeny

ILCs are a diverse family of immune cells with functions similar to T lymphocytes, including ILC1, ILC2, ILC3, natural killer (NK) cells, and lymphoid tissue inducer (LTi) cells [1–3]. Unlike T lymphocytes, ILCs do not possess antigen-specific receptors or undergo clonal selection and expansion. Instead, they respond to signals in their environment and regulate immune responses by specific cytokine secretion [2]. While the exact mechanisms of ILC differentiation are still not fully understood, it is widely accepted that ILCs originate from the common lymphoid progenitor (CLP) [4]. In mice, the CLP is characterized as Ly6D^−^, with the α4β7^+^ subset proposed as the earliest ILC progenitor, termed the α lymphoid progenitor (αLP) [5,6]. Additionally, the CLP gives rise to the early innate lymphoid progenitor (EILP), which differs from the αLP by lacking the interleukin-7 receptor (IL-7Rα, CD127) and the inhibitor of DNA binding 2 (Id2) [4,6]. Differentiation of both αLP and EILP is regulated by the transcription factor nuclear factor interleukin 3 regulated (NFIL3), which modulates Id2 expression [7,8]. Subsequent differentiation driven by transcription factor 7 (TCF-1) results in the formation of NK progenitors (NKP) and common helper-innate lymphoid cell progenitors (CHILP). This heterogeneous population includes both PLZF^+^ (zinc finger and BTB domain containing 16) and PLZF^−^ cells, which can differentiate into NK cells, ILC1, ILC2, ILC3, and LTis, respectively [9]. The terminal differentiation of these ILC subsets relies on specific transcription factors: T-bet and Eomesodermin (Eomes) for NK cells, T-bet for ILC1s, RORα and GATA3 for ILC2s and RORγt for ILC3s and LTis [4].

**Figure 1.**
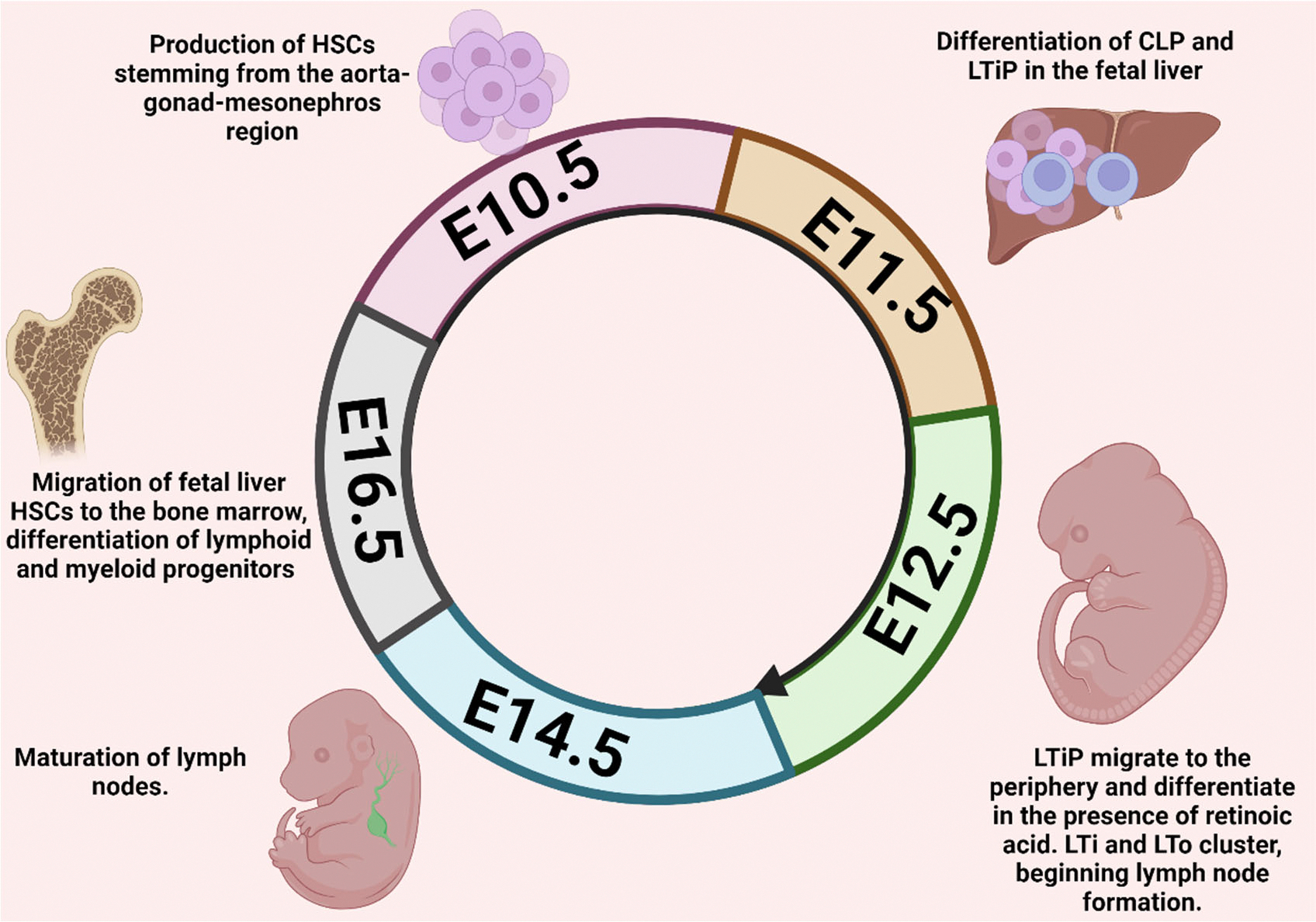
Ontogeny of embryonic ILC differentiation. LTi differentiation *in utero* occurs prior to the other ILC subsets and is required for the formation of lymph nodes. Following the migration to the fetal liver, HSCs differentiate into CLP and LTiP, then travel to the periphery. Exposure to retinoic acid allows for the transcription of RORγt, and subsequent maturation of LTi. LTis and LTos then cluster, which precedes the LTβR. HSC: Hematopoetic Stem Cell; CLP: Common Lymphoid Progenitor; LTi: Lymphoid Tissue Inducer; LTiP: Lymphoid Tissue Inducer Progenitor; LTo: Lymphoid Tissue Organizer. Figure created in BioRender.

To thoroughly address ILC ontogeny, it is essential to consider the distribution of CLPs during embryonic development, particularly in the differentiation of LTis. LTis were initially classified as a subset of ILC3s due to their similar transcriptomic profiles but have since been recognized as a distinct subset [4]. The differentiation of LTis *in utero* is believed to start in the aorta-gonad-mesonephros (AGM) region, where endothelial cells produce hematopoietic stem cells (HSCs) that subsequently migrate to the fetal liver (Figure 1) [10]. At embryonic day E16.5, fetal liver HSCs migrate to the bone marrow, where the majority of lymphoid differentiation occurs [10]. In contrast, the differentiation of LTis begins earlier in the fetal liver, where HSCs differentiate into CLP and LTi progenitor (LTiP) around E11.5. The LTiP then migrates to the periphery, a crucial step in LTi differentiation and the formation of lymph nodes (LNs) [10]. As the LTiP is exposed to retinoic acid in the periphery, the transcription of the retinoic acid-receptor-related orphan nuclear receptor gamma (RORγt) is induced [11]. The clustering of LTis and mesenchymal lymphoid tissue organizer (LTo) cells, essential for LN formation, occurs at E12.5. This clustering activates the lymphotoxin signaling pathway, leading to the maturation of LNs around E14.5. [10]. While most LTi differentiation in utero occurs in the periphery, a similar subset of LTis is also found in the fetal liver, where some acquire antigen-presenting capabilities through the expression of major histocompatibility complex (MHC)-II. This raises significant questions, particularly regarding whether the LTis observed in adults are true LTis or merely LTi-like cells that retain lymphotoxin signaling capacity. These differentiation divergences are not limited to LTis but extend to all ILC subsets. For instance, NK cells, which differentiate in the bone marrow, are unique in that they lack CD127 and thus develop in an IL-7-independent manner [12–14]. However, CD127^+^ NK cells have been identified in the LNs of mice, of which a percentage differentiate in the thymus [15]. Other tissue-resident (tr)NK cell subsets have also been identified in the liver, lung, and uterus, all of which demonstrate unique functions and phenotypes [16]. Similarly, cytotoxic intraepithelial ILCs are characterized by their absence of CD127, reliance on Eomes, and expression of CD103, an integrin that binds E-cadherin [17]. Distinct ILC2 and ILC3 subsets have also been described, such as the IL-10^+^ ILC2s, and the natural cytotoxicity receptor (NCR)^+^/NCR^−^ ILC3 subsets [18,19]. The variability in transcription factor activity and the environmental conditions during differentiation significantly contribute to the uncertainty surrounding ILC lineage commitment and the progenitors involved [20].

ILCs play distinct roles in modulating the immune response through their cytokine production. NK cells, known as the cytotoxic ILC subset and the innate counterparts to CD8^+^ T cells, produce inflammatory cytokines such as TNFα and IFNγ. They also mediate direct cytotoxicity by degrangulating, inducing target cell apoptosis, and performing antibody-dependent cell cytotoxicity [4]. ILC1s, generally thought to be the non-cytotoxic Th1 CD4^+^ counterpart, modulate inflammatory responses through their production of IFNγ [4]. However, a cytotoxic subset of ILC1s, termed “tumor-associated” ILC1s, has been found to express granzyme B and mediate NK cell-like cytotoxicity [21] ILC2s, the innate counterpart to Th2 CD4^+^ cells, modulate anti-inflammatory responses through their production of IL-5 and IL-13 [4,22]. ILC3s, the innate counterpart to Th17 CD4^+^ T cells, produce IL-17 and IL-22 and mediate gut immune responses [23].

Defining LTi cells and their functions is more complex due to their intricate ontogeny. LTi cells that form lymph nodes and differentiate in the periphery during embryogenesis are present for only a few weeks post-partum before being replaced by HSC-derived cells [10]. LTis that differentiate *ex utero*, known as LTi-like cells, develop in the bone marrow and express OX40L, CD30L, and LTα1β2 [24,25]. CXCL13 produced by both stromal cells and lymphocytes recruit LTis through CXCR5 signaling, inducing the formation of lymphoid structures, including the Peyer’s patches in the intestines [23,26]. During chronic inflammation and cancer, however, the recruitment of LTis to inflamed tissue can lead to the formation of ectopic tertiary lymphoid structures [27].

### The Dynamic and Multifaceted Roles of ILCs in Bladder Cancer

BCa carries one of the highest tumor mutational burdens, and the resulting immunogenicity makes it a great target for immunotherapies [28]. Current immunotherapies approved for use in BCa are immune checkpoint inhibitors (ICI), which aim to prevent T cell exhaustion [29]. The activation of cytotoxic CD8^+^ T cells requires antigen presentation by the target via the MHC-I, which is often downregulated by cancer cells as a mechanism of immune evasion, rendering T cell mediated cytotoxicity ineffective [30]. In contrast, innate immune cells like ILCs modulate immune activity independent of antigen specificity or presentation [4]. NK cells, for instance, mediate direct cell lysis by recognizing markers upregulated during cellular stress and detecting the downregulation of MHC-I [31]. Additionally, ILC1 and ILC3 cells produce pro-inflammatory cytokines, amplifying inflammatory responses [4].

In BCa, studies show that ILCs modulate both an inflammatory and immunosuppressive tumor microenvironment (TME) [4]. As we have previously discussed, NK cells compose a significant portion of bladder tumor-infiltrating lymphocytes and are crucial for anti-tumor activity [4]. In the BCa TME, however, NK cells are found to express various markers of exhaustion, including Tim-3 and PD-1, reducing their cytotoxic capacity [4]. ILC1s, which canonically contribute to modulating a pro-inflammatory immune environment, display a unique Th17-like phenotype in BCa, characterized by the production of IL-17, playing an overall pro-tumorigenic role [4,32].

ILC2s are known for their overall immunosuppressive nature, owing to their production of anti-inflammatory cytokines and chemokines such as IL-13 and CXCL2 [4,33,34]. Specifically, in the hypoxic TME, ILC2s have been found to produce IL-10, an immunosuppressive cytokine and negative regulator of inflammation [35–37]. In BCa, ILC2s have been correlated with increased infiltration of myeloid-derived suppressive cells (MDSCs), specifically M2-polarized macrophages [34]. The ILC2-modulated shift in the TME has also been correlated with the recurrence and progression of BCa. Recently, however, an *in vivo* BCa model demonstrated that inhibiting or depleting ILC2s does not affect mouse survival or tumor development. Nonetheless, their exact contribution to BCa progression warrants further investigation [33].

The role of the ILC3 subset in BCa remains underexplored, but their highly plastic nature suggests they could have both beneficial and detrimental effects. As previously discussed, ILC1s in BCa have exhibited an ILC3-like phenotype and were found to accumulate in higher-stage tumors [32]. Additionally, the co-expression of CD69 and CD103, along with elevated levels of CCR6, on ILC3s contribute to increased recruitment to lymphoid tissue and tissue residency in BCa [4,38]. These LTi-like ILC3s are particularly interesting due to their association with tertiary lymphoid structure (TLS) formation [39].

**Figure 2.**
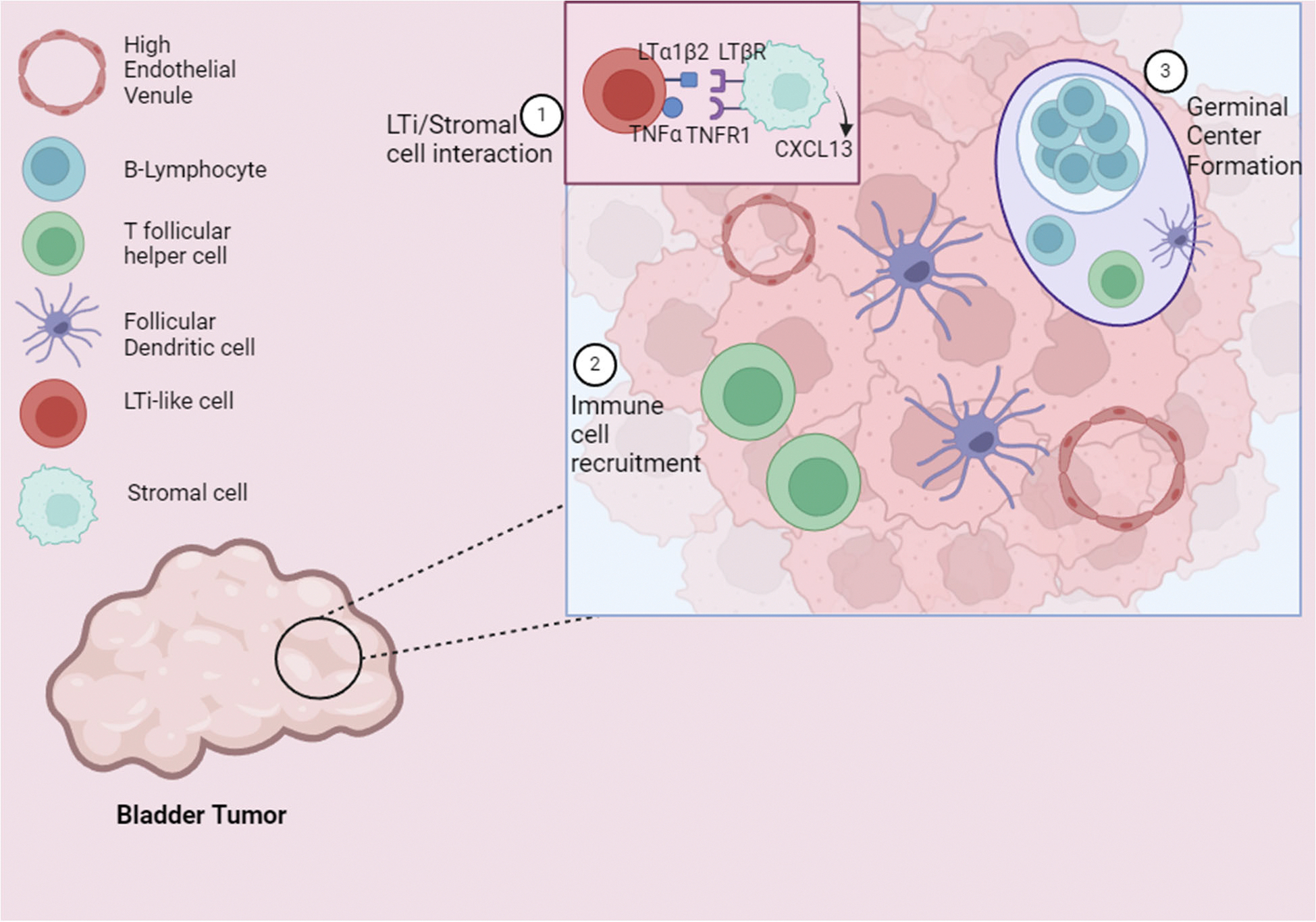
LTi-like cells interact with stromal cells and induce the formation of TLSs in BCa. LTi-like cells respond to signaling chemokines produced by stromal cells in BCa via the CXCL13/CXCR5 axis. The stromal cells expressing LTβR and TNFR1 then bind and interact with LTα1β2 and TNFα expressed by the LTi, which produce cytokines that induce the formation of high endothelial venules and recruit B cells, T follicular helper cells, and follicular dendritic cells, which form germinal centers in the TLS. LTi: Lymphoid Tissue Inducer; TLS: Tertiary Lymphoid Structure; LTβR: Lymphotoxin Β Receptor; TNFα: Tumor Necrosis Factor α; LTα1β2: Lymphotoxin α1β2; TNFR1: Tumor Necrosis Factor Receptor. Figure created in BioRender.

LTis are responsible for the formation of TLSs (Figure 2), ectopic aggregates of immune cells resembling secondary lymphoid organs (SLO) [26,40]. The binding of the lymphotoxin (LT)α1β1 expressed on LTi to the LTβR on stromal cells induces the release of chemokines including CXCL13, CXCL12, CCL21, and CCL19, which further recruit CD4^+^ T cells, follicular dendritic cells (FDC), and B cells [39]. Mature TLSs are structured similarly to SLO, with CD3^+^ T cell-rich areas as well as FDC and T follicular helper cells (Tfh) capable of forming germinal centers [39]. TLSs are becoming increasingly relevant in cancer due to their role in enhancing lymphocyte recruitment and maturation. However, in some malignancies, they have also been found to play an immunosuppressive role [41]. TLSs in renal cell carcinoma increased the population of tumor-specific IgG-producing plasma cells due to their *in situ* hypermutation and clonal expansion capabilities [42]. In BCa, mature TLSs correlated with increased immune infiltration and improved patient prognosis [42]. Additionally, TLSs expressing higher levels of CXCL13 were associated with better BCa outcomes and an improved response to ICI therapy, suggesting that CXCL13 is a possible biomarker for disease progression and ICI therapy effectiveness [42]. Due to the nature of BCa, biomarkers relating to ILC infiltration are difficult to quantify without invasive procedures, such as biopsies. For this reason, it is pertinent to define additional urinary biomarkers which can be used to screen for ILC infiltration.

It is evident that ILCs play a vital role in BCa prognosis, but the extent of their impact remains unclear due to limited research. While targeting cytotoxic ILC subsets appears to be a promising strategy for enhancing anti-tumor immunity, the plasticity of non-cytotoxic ILCs may also play a crucial role [43]. Evidence of ILC2 plasticity has emerged from studies in other malignancies. For instance, in hepatocellular carcinoma, a higher ILC2-to-ILC1 ratio with a unique KLRG1^+^ phenotype correlated with a better prognosis compared to KLRG^−^CXCL8^hi^ ILC2s, which are known to promote the recruitment of immunosuppressive neutrophils [18]. Interestingly, an inflammatory ILC2 subset emerged following the reconstitution of allogeneic HSC transplantation, in response to inflammatory cytokines. [44]. Incubation of ILC2s with inflammatory cytokines *in vitro* also induced an inflammatory phenotype, as evidenced by changes in the transcriptome and chromatin modifications of STAT1 and IFNγ [44].

### ILCs in Bladder Cancer Prognosis and Treatment–Past, Present, and Future

The downregulation of MHC-I as an immune evasion mechanism has been correlated with immune therapy failure and recurrence of BCa [45]. Due to the inhibitory mechanism of NK cells, the downregulation of MHC-I increases BCa susceptibility to NK cytotoxicity [4]. The adoptive transfer of NK cells is a promising therapeutic strategy in BCa due to their ability to mediate tumor lysis independently of antigen presentation [46]. However, its clinical efficacy is limited by several factors, including the exhaustion of transferred NK cells, which diminishes their cytotoxic function, and insufficient tumor infiltration. Additionally, the immunosuppressive tumor microenvironment, characterized by inhibitory cytokines, metabolic constraints, and physical barriers, further impairs NK cell persistence, infiltration, and antitumor activity, ultimately contributing to the suboptimal outcomes of adoptive NK cell therapy [47]. Current treatments targeting NK cells include immunomodulatory drugs, NK receptor agonists, interleukins, and chimeric antigen receptor (CAR)-expressing NK cells (Table 1) [48]. Lenalidomide, vactosertib, and durvalumab are three immunomodulatory drugs currently undergoing clinical trials for BCa. Lenalidomide (NCT01373294, NCT01342172, NCT01352962) functions as an NK cell activator by stimulating T cell production of IL-2 and IFNγ while vactosertib and durvalumab (NCT04064190) inhibit the TGFβ type 1 receptor kinase, inhibiting TGFβ-induced NK cell downregulation [48–52]. NK receptor agonists currently being tested target the co-receptors CD27, OX40, GITR, and 4–1BB [48,53,54]. Both OX40 and 4–1BB are also expressed in T cells, allowing these agonists to target both the innate and adaptive immune populations [48,55]. Other combination therapies targetting adaptive and innate immunity are also being tested in BCa, such as the combination of the anti-PD-1 drug, nivolumab, and anti-KIR2DL lirilumab in cisplatin-refractive muscle-invasive BCa (NCT0353245) [56]. Interleukin administration is another treatment that targets NK cells as well as other inflammatory immune cells [48]. For example, IL-2, an inflammatory cytokine required for NK cell proliferation, cytotoxicity, and survival, immunotherapy was approved nearly 20 years ago and remains a treatment choice as both a monotherapy and combination therapy in multiple metastatic cancers [57,58].

Alternative drug approaches targeting NK cells in BCa remain in the preclinical phase but show promise [59]. Recently, the anti-androgen drug enzalutamide demonstrated increased efficacy in mediating NK cell cytotoxicity both *in vitro* and *in vivo* [60]. Liu *et al.* demonstrated that signaling through the androgen receptor modulated the adenosine deaminase acting on RNA (ADAR) 2 enzyme, leading to decreased miR-200–3p via circ_0001005 sponging and resulting in increased PD-L1 expression in BCa [60]. Treatment of BCa cells with androgen-targeting drugs, including the FDA-approved prostate cancer treatment Enzalutamide, enhanced NK cell cytotoxicity by downregulating PD-L1 expression [60].

In an orthotopic murine model of BCa, treatment with a CD39 inhibitor significantly reduced tumor burden and increased infiltration of cytotoxic T lymphocytes (CTLs), NK cells, and conventional dendritic cells (cDCs), leading to enhanced CTL proliferation [61]. However, this anti-tumor effect was lost following NK cell depletion, highlighting the crucial role of NK cells in recruiting both cDCs and CTLs [61]. Additionally, Huyan et al. recently showed the impact of tumor-derived exosomes on NK cells. Exosomes released by the BCa cell line T24 induced NK cell apoptosis and downregulated the expression of DAP10, perforin, and CD96 through the delivery of microRNAs miR-221–5p and miR-186–5p [62].

Drug therapies targeting ILCs beyond NK cells remain limited due to sparse research on these subsets. However, existing studies are promising and warrant further investigation. For example, in an orthotopic model of pancreatic ductal adenocarcinoma, deleting IL-33 led to increased tumor burden due to reduced recruitment of ILC2s [50]. Conversely, treatment with recombinant IL-33 and anti-PD-1 decreased tumor burden, with IL-33 stimulation enhancing the sensitivity of ILC2s to anti-PD-1 therapy [63]. Additionally, stimulating NCR^+^ILC3s with IL-12 can activate the inflammatory cascade, suggesting that recombinant IL-12 treatment could be a viable therapeutic strategy [64]. These findings draw valuable lessons from other solid tumors and highlight the potential for targeting ILCs to enhance BCa treatment. However, special consideration is required when targeting ILCs due to their tissue-specific behavior. For instance, while recombinant IL-33 treatment reduces tumor burden in pancreatic ductal adenocarcinoma, in BCa, IL-33 may exacerbate the ILC2-mediated recruitment and polarization of MDSCs.

As research on ILCs advances, the heterogeneity within ILC subsets becomes increasingly evident. We have highlighted instances of ILC plasticity in BCa, such as the ILC3-like phenotype observed in ILC1s [32]. However, it remains unclear whether these phenotypic shifts are driven by the TME or are specific to bladder tissue-resident ILCs. To effectively target ILC subsets in BCa, it is essential to fully characterize the unique phenotype and function of tissue-resident ILCs. Additionally, the role of the bladder TME in shaping ILC differentiation and recruitment remains poorly understood and warrants further investigation. Identifying the signals that drive ILC differentiation may shed light on the mechanisms behind their skewed functionality in BCa. For example, in chronic obstructive pulmonary disease and asthma, upregulation of inflammatory cytokines IL-1β and IL-12 promotes the differentiation of ILC2s into ILC1s, leading to chronic inflammation and tissue damage [65]. Understanding whether similar mechanisms occur in BCa could inform therapeutic strategies, such as leveraging the ILC2 axis in recombinant IL-12 treatment.

Despite the established importance of ILCs, the ontogeny, residency, and phenotypic expression of ILCs in BCa remain largely unexplored. To develop effective treatments targeting ILCs, a thorough understanding of their functions and roles within BCa is imperative. Single-cell RNA sequencing technologies allow for precise phenotypic characterization of tissue-specific ILCs across various tissues. When combined with *in vivo* infection and cancer models, this approach can elucidate the plasticity of tissue-resident ILCs in disease. Additionally, chromatin immunoprecipitation sequencing (ChIP-seq) may offer a comprehensive view of ILCs in cancer, paving the way for the development of novel ILC-targeting therapies tailored to the site of malignancy. Lastly, advancing BCa treatment requires integrating ILC-targeted strategies into clinical research. This involves incorporating ILC profiling into clinical trials to correlate ILC subsets with treatment outcomes and exploring ILC modulation or depletion as potential adjuvant therapies. By embedding ILC-focused approaches into both preclinical and clinical studies, we can uncover strategies to overcome immunotherapy resistance and fully realize the potential of ILC-targeted therapies. To fully realize these benefits, identifying specific biomarkers to guide therapy choice is also essential. Embracing precision medicine will allow for the customization of treatments to individual patients, thereby enhancing their effectiveness and advancing overall treatment strategies in BCa.

## Figures and Tables

**Table 1.** Current NK targeting immunotherapies in clinical trials.

Therapy	Functional Target	Approval Status	Ref
ALT-803/N-803	IL-15 agonist	Awaiting FDA approval for use in high risk NMIBC	[66,67]
CG0070	GM-CSF expressing oncolytic adenovirus	Phase III	[68,69]
Durvalumab	TGFβ inhibitor	FDA approved for biliary malignancy, lung cancer (small cell and non-small cell), and hepatocellular carcinoma. Phase II trial for efficacy in bBCa as an adjuvant	[70,71]
Lenalidomide	Stimulates production of IFNγ and IL-2 by T cells	Approved for multiple myeloma, specific lymphomas. Phase Ib/II trials for metastatic BCa in combination with gemcitabine and cisplatin.	[50–52,72]
MEDI6469 MOXR0916 INBRIX-106	Anti-OX40 antibody; OX40 agonist	Terminated Terminated Phase 1 / 2 Trials	[73–76]
NK-CD24-CAR cell therapy	Targets highly expressed CD24 in BCa to activate CAR-NK cells	Pre-Clinical	[77]
Proleukin	Synthetic IL-2 treatment	FDA approved in melanoma and renal carcinoma	[78]
TRX518	Glucocorticoid-induced TNF receptor-related protein agonist	Phase 1b for use in solid tumors	[79]
Urelumab Utomilumab	CD137 (4–1BB) Agonist	Awaiting FDA Approval; Phase II/dose escalation	[80,81]; [82]
Vactosertib	TGFβ receptor inhibitor	Phase 1b for use in multiple myeloma, as well as in combination with durvalumab in urothelial carcinoma	[49,83,84]
Varlilumab	CD27 targetting monoclonal antibody	Phase II for various solid tumors, as well as in combination with anti-PD-1 therapy	[53,54,85]
Nivolumab and Lirilumab	Anti-PD-1 and anti-KIR2DL combination therapy	Phase 1b for MIBC ineligible for cisplatin-based therapy	[56]

NMIBC: Non-Muscle Invasive Bladder Cancer; MIBC; Muscle-Invasive Bladder Cancer; IL: Interleukin; GM-CSF: Granulocyte-Macrophage Colony-Stimulating Factor; CAR-NK: Chimeric Antigen Receptor Natural Killer; TGFβ Transforming Growth Factor β; IFNγ: Interferon γ; TNF: Tumor Necrosis Factor; OX40: Tumor Necrosis Factor Receptor Superfamily Member 4; KIR2DL: Killer-Cell Immunoglobulin-Like Receptor Two Ig Domains and Long Cytoplasmic Tail.
